# Inpatient mortality and associated clinical factors among people living with HIV with cryptococcal meningitis in Uganda: A retrospective cohort study

**DOI:** 10.1371/journal.pone.0340951

**Published:** 2026-06-26

**Authors:** Joshua Kitimbo, Esther Buregyeya, Geofrey Mutole, John Paul Ibanda, Joseph Tumwine, Noah Kiwanuka, Thomas Buyinza

**Affiliations:** 1 Department of Community Health and Behavioural Sciences, School of Public Health, Makerere University, Kampala, Uganda; 2 Department of Disease Control and Environmental Health, School of Public Health, Makerere University, Kampala, Uganda; 3 Department of Health Policy Planning and Management, School of Public Health, Makerere University, Kampala, Uganda; 4 College of Veterinary Medicine, Animal Resources and Bio-Security, Makerere University, Kampala, Uganda; 5 Department of Epidemiology and Biostatistics, School of Public Health, Makerere University, Kampala, Uganda; 6 Iowa State University-Uganda Program, Center for Sustainable Rural Livelihoods, Kamuli, Uganda; Mansoura University Faculty of Veterinary Medicine, EGYPT

## Abstract

**Introduction:**

Cryptococcal meningitis remains a leading cause of HIV-related mortality in sub-Saharan Africa despite expanded antiretroviral therapy coverage. Evidence on the burden of disease and inpatient mortality among people living with HIV (PLHIV) in routine care settings in Uganda remains limited. This study assessed the proportion of cryptococcal meningitis among HIV-related admissions and examined clinical factors associated with inpatient mortality.

**Methods:**

We conducted a retrospective cohort study of adult PLHIV admitted with cryptococcal meningitis between 1^st^/01/2017–31^st^/12/2022 at a national referral hospital in Uganda. Diagnosis was based on cerebrospinal fluid cryptococcal antigen or India ink positivity. Data were abstracted from medical records and analysed using descriptive statistics and multivariable logistic regression to identify factors associated with inpatient mortality. Specific antifungal treatment regimens were not consistently documented and could not be analysed.

**Results:**

Of 3,042 HIV-related admissions, cryptococcal meningitis accounted for 21.4% (650/3,042). Medical records for 634 patients were analysed, among whom 39.3% (249/634) died during hospitalization. Factors independently associated with higher odds of inpatient mortality included convulsions, headache, vomiting, cryptococcal meningitis–associated immune reconstitution inflammatory syndrome, concurrent opportunistic infections, chronic kidney disease, anaemia, and severe immunosuppression (low CD4 cell count). Longer duration of hospitalization (≥7 days) and symptom duration of one to two weeks before admission were associated with lower odds of mortality.

**Conclusion:**

Cryptococcal meningitis continues to account for a substantial proportion of HIV-related hospital admissions and inpatient deaths in Uganda. Mortality is associated with identifiable clinical and health-system factors, underscoring the need for early diagnosis, risk stratification, and optimized inpatient management for PLHIV with cryptococcal meningitis in resource-limited settings.

## Introduction

Cryptococcal meningitis (CM) is a life-threatening opportunistic fungal infection of the central nervous system that occurs predominantly among people living with HIV (PLHIV) with advanced immunosuppression. It is caused mainly by Cryptococcus neoformans and less frequently by Cryptococcus gattii, with infection typically acquired through inhalation of environmental spores followed by hematogenous dissemination to the brain, resulting in severe meningoencephalitis [1]. Without timely diagnosis and effective antifungal treatment, cryptococcal meningitis is associated with rapid clinical deterioration, neurological complications, and high short-term mortality [2,3].

Despite substantial progress in HIV care and expanded access to antiretroviral therapy (ART), cryptococcal meningitis remains a major cause of HIV-related morbidity and mortality globally, with the greatest burden concentrated in sub-Saharan Africa (SSA) [4]. Of the estimated 38.4 million PLHIV worldwide, more than two-thirds reside in SSA [5], where late HIV diagnosis, advanced disease at presentation, and limited access to optimal management of opportunistic infections persist [6]. Cryptococcal meningitis accounts for over 70% of cryptococcal disease among HIV-infected individuals and is responsible for approximately 20% of HIV-related deaths globally, highlighting its continued contribution to preventable mortality [7,8].

In Uganda, CM is the leading cause of adult HIV-associated meningitis and remains a major contributor to inpatient morbidity, mortality, and long-term neurological disability among PLHIV [9]. Although national and international guidelines recommend routine cryptococcal antigen (CrAg) screening and standardized antifungal therapy for individuals with advanced HIV disease, implementation gaps remain common [7,10]. Limited access to rapid diagnostics, inconsistent availability of amphotericin-based regimens, inadequate monitoring and management of raised intracranial pressure, and delayed care-seeking continue to undermine survival outcomes in routine hospital settings [3,11].

Clinical factors such as severe immunosuppression, anaemia, seizures, concurrent opportunistic infections, renal dysfunction, and cryptococcal meningitis–associated immune reconstitution inflammatory syndrome (CM-IRIS) have consistently been associated with increased risk of inpatient mortality [12–14]. However, much of the existing evidence is derived from clinical trials, short-term cohorts, or mixed inpatient–outpatient populations, which may not adequately reflect routine care conditions in high-burden, resource-limited settings [4,15]. In Uganda, contemporary evidence quantifying the burden of cryptococcal meningitis among HIV-related admissions and systematically examining inpatient mortality and its correlates over extended periods remains limited.

To address this gap, we analysed six years of routinely collected inpatient data from Kiruddu National Referral Hospital (KNRH) to quantify the proportion of cryptococcal meningitis among HIV-related admissions and to examine clinical and health-system factors associated with inpatient mortality. Generating such real-world evidence is critical for informing risk stratification, strengthening inpatient management, and guiding targeted interventions to reduce preventable deaths among PLHIV with cryptococcal meningitis in resource-limited settings.

## Methods

### Study design and setting

We conducted a retrospective cohort study using routinely collected medical records of adult PLHIV admitted with cryptococcal meningitis at Kiruddu National Referral Hospital (KNRH) in Kampala, Uganda, from 1 January 2017–31 December 2022. KNRH provides specialised inpatient care for HIV-related opportunistic infections, including cryptococcal meningitis and tuberculosis, and receives referrals from across Uganda.

### Study population and eligibility criteria

The study population comprised adult PLHIV whose medical records documented HIV-associated cryptococcal meningitis during the study period. ART-naïve and ART-experienced patients were eligible, including both referral and non-referral cases. Records were excluded if they lacked documented inpatient outcomes, involved HIV-negative patients diagnosed with cryptococcal meningitis, or were unavailable for review.

### Sampling procedure

A total population sampling approach was used. All available medical records of eligible adult PLHIV admitted with cryptococcal meningitis during the study period were consecutively reviewed to maximise statistical power and minimise selection bias. Of 3,042 HIV-related admissions, 650 cryptococcal meningitis cases were identified; records for 634 patients (97.5%) were successfully retrieved and included in the final analysis.

### Data abstraction and quality assurance

Data were abstracted from hard-copy medical records using a structured abstraction tool programmed in Kobo Collect. Medical records were accessed, retrieved, and abstracted between 12 December 2022 and 31 January 2023. The tool was developed based on a review of published literature on predictors of cryptococcal meningitis mortality and relevant clinical guidelines. Variables captured included sociodemographic characteristics, clinical presentation, laboratory findings, comorbidities, and in-hospital outcomes.

The abstraction tool underwent expert review by clinicians experienced in HIV and cryptococcal meningitis management and by epidemiologists familiar with retrospective cohort studies. Trained research assistants conducted data abstraction using mobile devices, following standardized procedures. Data quality was ensured through comprehensive training, pilot testing of the abstraction tool, and daily supervisory checks for completeness and internal consistency.

Cryptococcal meningitis was defined by a positive cerebrospinal fluid cryptococcal antigen (CrAg) test or India ink microscopy, while, cryptococcal meningitis–associated immune reconstitution inflammatory syndrome (CM-IRIS) was retrospectively defined based on documented positive CSF CrAg, clinical deterioration (e.g., worsening headache, fever, photophobia, or convulsions), and occurrence within 2–10 weeks following initiation of antiretroviral therapy. CD4 T cell count was categorised into three groups: < 50 cells/µL, 50–200 cells/µL, and >200 cells/µL, based on clinical relevance and prior literature on advanced HIV disease. Data on antifungal treatment regimens were not consistently documented in medical records and were therefore not included in the analysis.

### Data management

Data were exported from Kobo Collect to Microsoft Excel for cleaning and management and subsequently analysed using Stata version 15. Data were checked for completeness and consistency prior to analysis. Missing data were assessed for all variables included in the analysis. No missingness was observed (**S1 File**); therefore, complete-case analysis was conducted.

### Statistical analysis

Descriptive statistics were used to summarize patient characteristics, with categorical variables presented as frequencies and percentages and continuous variables summarized using means and standard deviations or medians and interquartile ranges, as appropriate. The proportion of cryptococcal meningitis among HIV-related admissions was calculated as the percentage of confirmed cases relative to all PLHIV admissions. In-hospital mortality was defined as death occurring during the admission in which cryptococcal meningitis was diagnosed.

Associations between explanatory variables and inpatient mortality were examined using logistic regression, as reliable time-to-event data were not consistently available to support survival analysis. Bivariate logistic regression analyses were initially conducted, and variables with p ≤ 0.20 were considered for inclusion in the multivariable model. Multicollinearity among candidate independent variables was assessed using variance inflation factors (VIF), with values greater than 10 considered indicative of problematic collinearity; no evidence of multicollinearity was detected (all VIF values were below 10, with a mean VIF of 1.10). A multivariable logistic regression model was subsequently fitted using a forward stepwise approach. Statistical significance was set at p ≤ 0.05, and results are presented as adjusted odds ratios (AORs) with 95% confidence intervals.

### Ethical considerations

Ethical approval was obtained from the Makerere University School of Public Health Higher Degrees Research and Ethics Committee (study protocol number 131), with a waiver of informed consent granted for retrospective review of de-identified medical records. Permission to access patient records was granted by KNRH. Data abstractors had no access to patient records that could identify them during and after data abstraction. All data were anonymised prior to analysis to ensure confidentiality.

## Results

### Patient admissions and records retrieval

**Fig 1:**
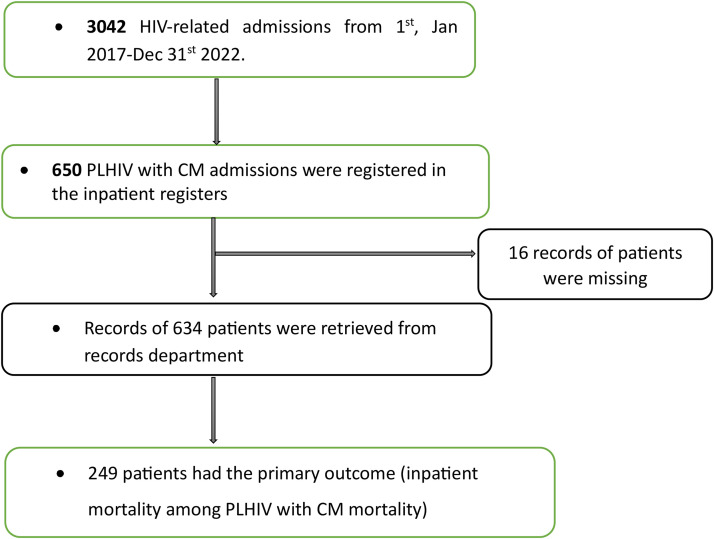
Flow diagram of patient selection and inclusion in the study.

Between January 2017 and December 2022, a total of 3,042 HIV-related inpatient admissions were recorded at KNRH in Uganda (**Fig 1**). Among these admissions, 650 patients (21.4%) were diagnosed with HIV-associated cryptococcal meningitis according to inpatient registers. Medical records for 634 of these patients (97.5%) were successfully retrieved and included in the analysis. Sixteen records (2.5%) could not be retrieved due to incomplete documentation and were excluded.

Of the 634 patients included, 249 died during hospitalization, corresponding to an overall in-hospital mortality of 39.3%.

### Socio-demographic and clinical characteristics

The median age of participants was 36 years (IQR = 15), with the majority aged 30–39 years (38.0%) (Table 1); 52.2% were male. Symptom duration prior to admission was evenly distributed, with 31.1% presenting within one week, 37.4% within one to two weeks, and 31.5% after more than two weeks.

**Table 1:** Socio-demographic and clinical characteristics of the patients in the study.

Parameter	Frequency (n = 634)	Percentage (%)
**Age (years), median = 36 (IQR = 15)**		
<20	9	1.4
20–29	140	22.1
30–39	241	38.0
40–49	146	23.0
50–59	64	10.1
60+	34	5.4
**Sex**		
Male	331	52.2
Female	303	47.8
**Duration of hospitalization (days), median = 6 (IQR = 10)**		
<7 days	319	50.3
7–14 days	198	30.2
>14 days	117	18.5
**Duration of symptoms before hospitalization**		
Less than 1 week	197	31.1
1–2 weeks	237	37.4
Greater than 2 weeks	200	31.5
**Duration of ART**		
ART-naive	97	15.3
Less than 1 year	206	32.5
1–5 years	223	35.2
Greater than 5 years	108	17.0
**Previous history of cryptococcal meningitis**		
Yes	222	35.0
No	412	65.0
**Signs, symptoms and comorbidities present at the time of diagnosis**		
Headache	553	87.2
Vomiting	372	58.7
Blurring of vision	151	23.8
Cryptococcal meningitis–associated IRIS	73	11.5
Concurrent opportunistic infection(s)	299	47.2
**Specific opportunistic infections documented**		
Tuberculosis	183	28.9
Pneumonia	39	6.2
Oral and/or esophageal candidiasis	27	4.3
Sepsis	16	2.5
Kaposi sarcoma	14	2.2
Other opportunistic infections	96	15.1
Concurrent diabetes mellitus	30	4.7
Concurrent cardiovascular disease or hypertension	26	4.1
Concurrent chronic kidney disease	81	12.1
**Hemoglobin level (g/dL), mean (SD) = 9.6 (3.3)**		
<8	223	35.2
8–10	163	25.7
10.1–12.5	109	17.2
>12.5	139	21.9
**CD4 T cell count (cells/µL), median = 45 (IQR = 89)**		
<50	333	52.5
50–200	257	40.6
>200	44	6.9

***Note:*** *Percentages are calculated using N = 634. Specific opportunistic infection categories may not be mutually exclusive where more than one infection was documented.*

Headache was the most common presenting symptom (87.2%), followed by vomiting (58.7%) and blurred vision (23.8%). A previous history of cryptococcal meningitis was reported in 35.0% of patients. The median hospital stay was 6 days (IQR = 10), with half (50.3%) admitted for fewer than seven days.

Laboratory findings indicated advanced immunosuppression (median CD4 = 45 cells/µL; IQR = 89), with 52.5% having CD4 < 50 cells/µL and only 6.9% > 200 cells/µL. Anaemia was common (mean haemoglobin 9.6 g/dL; SD = 3.3). Nearly half (47.2%) had at least one opportunistic infection, predominantly tuberculosis.

### Burden of cryptococcal meningitis and inpatient mortality

Cryptococcal meningitis accounted for 21.4% (650/3,042) of all HIV-related inpatient admissions during the study period. Among patients admitted with cryptococcal meningitis and included in the analysis, 39.3% (249/634) died during hospitalization.

### Factors associated with inpatient mortality among PLHIV with cryptococcal meningitis

At the bivariate level, several clinical and laboratory factors were associated with inpatient mortality at p ≤ 0.20 (Table 2). Longer duration of hospitalization was associated with lower odds of death, with patients hospitalized for 7–14 days (crude odds ratio [COR] = 0.66; 95% CI: 0.46–0.95) and more than 14 days (COR = 0.57; 95% CI: 0.36–0.89) having reduced mortality compared with those hospitalized for fewer than seven days.

**Table 2:** Clinical factors associated with inpatient mortality among PLHIV with cryptococcal meningitis at a national referral hospital in Uganda, between 2017 and 2022.

Variable	Inpatient mortality		COR (95%CI)	AOR (95% CI)
	Yes (%)	No (%)		
**Sex**				
Female Males	111 (36.6) 138 (41.7)	192 (63.37) 193 (58.31)	1 **1.24** [0.90-1.71]	‒
**Age (years)**				
60+ <20 20-29 30-39 40-49 50-59	18 (53.9) 4 (44.4) 49 (35.0) 90 (37.3) 64 (43.8) 27 (42.2)	16 (47.1) 5 (55.6) 91 (65.0) 151 (62.7) 82 (56.2) 37 (57.8)	1 1.54 [0.30-2.56] 0.48 [0.22-1.12] 0.49 [0.24-1.20] 0.69 [0.33-1.47] 0.64 [0.28-1.50]	‒
**Duration of hospitalization (days)**				
<7 7-14 >14	142 (45.5) 67 (33.8) 37 (31.6)	177 (55.5) 131 (66.2) 80 (68.4)	1 0.66 [0.46-0.95] 0.57 [0.36-0.89]	1 **0.58** [0.37-0.88] **0.49** [0.30-0.85]
**Duration of symptoms before:**				
<1 week 1-2 weeks >2 weeks	90 (45.7) 60 (25.3) 96(48.0)	107 (54.3) 177(74.7) 104(52.0)	1 **0.41** [0.28-0.61] 1.11 [0.77-1.69]	1 **0.41** [0.26-0.65] 1.10 [0.71-1.72]
**Duration of HAART**				
ART naïve Less than 1 year 1-5 years Greater than 5 years	45 (46.4) 79 (38.3) 85 (38.1) 40 (37.0)	52 (53.6) 127 (61.7) 138 (61.9) 68 (63.0)	1 0.72 [0.44-1.17] 0.71 [0.44-1.15] 0.71 [0.41-1.25]	‒
**Previous history of CM**				
No Yes	160(41.5) 78 (35.1)	241 (58.5) 144 (64.9)	1 1.30 [0.66-1.29]	
**Convulsions**				
Absent Present	187 (36.2) 62 (47.5)	329 (63.8) 56 (52.5)	1 1.94 [1.30-2.91]	1 **1.63** [1.03-2.56]
**Headache**				
No Yes	62 (76.5) 230 (41.0)	19 (23.5) 323 (59.0)	1 **2.32** [1.35-3.99]	1 **2.08** [1.13-3.85]
**Vomiting**				
No Yes	86 (32.8) 163 (43.8)	176 (67.2) 209 (56.2)	1 1.59 [1.17-2.28]	1 **1.61** [1.10-2.36]
**Blurring of vision**				
No Yes	184 (38.1) 65 (43.1)	299 (61.9) 86 (56.9)	1 1.22 [0.85-1.78]	‒
**CM- IRIS**				
No Ye**s**	198 (35.3) 51 (69.9)	363 (64.7) 22 (30.1)	1 **4.25** [2.50-7.21]	1 **3.96** [2.22-7.05]
**Concurrent Opportunistic infections**				
No Yes	113 (33.7) 136 (45.5)	222 (66.3) 163 (54.5)	1 **1.63** [1.23-2.36]	1 **1.56** [1.08-2.26]
**Concurrent DM** Absent Present	237 (38.7) 12 (40.0)	367 (61.3) 18 (60.0)	1 1.03 [0.50-2.23]	‒
**Concurrent CVD**				
Present Absent	240 (39.5) 9 (34.6)	368 (60.5) 17 (65.4)	1 0.81[0.36-1.85]	‒
**Concurrent CKD**				
Present Absent	347(62.7) 43 (53.1)	206 (37.3) 38 (46.9)	1 **1.90** [1.15-2.94]	1 **1.77** [1.05-2.99]
**Hemoglobin (g/dl)**				
>12.5 <8 Hb 8–10 10.1-12.5	41 (70.5) 101 (45.3) 71 (43.6) 36 (33.0)	98 (29.5) 122 (54.7) 92 (56.4) 73 (67.0)	1 1.99 [1.28-3.16] 1.84 [1.15-3.01] 1.17 [0.71-2.10]	1 **2.08** [1.25-3.44] **1.87** [1.10-3.18] 1.23 [0.67-2.34]
**CD4 T cell count (cell/uL)**				
<50 50-200 >200	145 (43.3) 95 (37.2) 9 (20.4)	190 (56.7) 160(62.8) 35 (79.6)	1 0.78[0.55-1.08] 0.34[0.26-0.72]	1 **0.34** [0.12-0.62] **0.27** [0.14-0.78]

*Note: AORs are shown only for variables retained in the final multivariable model. Reference categories are indicated by 1*

Symptom duration before admission of one to two weeks was associated with lower odds of death compared with presentation within one week (COR = 0.41; 95% CI: 0.28–0.61). Clinical features associated with higher mortality included convulsions, headache, vomiting, cryptococcal meningitis–associated immune reconstitution inflammatory syndrome (CM-IRIS), concurrent opportunistic infections, chronic kidney disease, anaemia, and CD4 cell count category.

After adjustment for potential confounders, several factors remained independently associated with inpatient mortality. Longer hospitalization was associated with reduced odds of death, including stays of 7–14 days (adjusted odds ratio [AOR] = 0.58; 95% CI: 0.37–0.88) and more than 14 days (AOR = 0.49; 95% CI: 0.30–0.85). Presentation after one to two weeks of symptoms was also associated with lower mortality (AOR = 0.41; 95% CI: 0.26–0.65). Clinical factors independently associated with increased odds of death included convulsions (AOR = 1.63; 95% CI: 1.03–2.56), headache (AOR = 2.08; 95% CI: 1.13–3.85), vomiting (AOR = 1.61; 95% CI: 1.10–2.36), CM-IRIS (AOR = 3.96; 95% CI: 2.22–7.05), concurrent opportunistic infections (AOR = 1.56; 95% CI: 1.08–2.26), and chronic kidney disease (AOR = 1.77; 95% CI: 1.05–2.99).

Anaemia remained strongly associated with mortality. Compared with patients with haemoglobin >12.5 g/dL, those with haemoglobin <8 g/dL (AOR = 2.08; 95% CI: 1.25–3.44) and 8–10 g/dL (AOR = 1.87; 95% CI: 1.10–3.18) had higher odds of death. CD4 cell count categories were also associated with inpatient mortality, with AORs of 0.34 (95% CI: 0.12–0.62) for 50–200 cells/µL and 0.27 (95% CI: 0.14–0.78) for >200 cells/µL.

## Discussion

This study assessed inpatient mortality and associated clinical factors among adult PLHIV with cryptococcal meningitis admitted to a national referral hospital in Uganda between 2017 and 2022. Cryptococcal meningitis accounted for more than one-fifth of HIV-related inpatient admissions, and in-hospital mortality was high (39.3%). Mortality was associated with clinical severity, including neurological symptoms, CM-IRIS, anaemia, low CD4 cell count, and concurrent opportunistic infections. These findings provide real-world evidence on the burden and clinical correlates of cryptococcal meningitis outcomes in routine inpatient care in a high-burden setting.

The observed burden and mortality remain substantial and are consistent with reports from similar settings in sub-Saharan Africa [3,15].The concentration of mortality among young adults reflects a considerable burden in economically productive populations [16]. However, mortality appears to be more strongly influenced by clinical severity and comorbidity than by age alone, underscoring the importance of timely diagnosis and effective inpatient management.

Clinical presentation was strongly associated with mortality. Symptoms such as headache, convulsions, and vomiting likely reflect raised intracranial pressure, a key predictor of poor outcomes [3,11]. The lower mortality observed among patients presenting after one to two weeks of symptoms should be interpreted cautiously, as it may reflect differences in disease progression, survival to admission, or care-seeking patterns rather than a protective effect of delayed presentation. Limited documentation of intracranial pressure in this study suggests potential gaps in routine monitoring and adherence to recommended management practices in similar resource-limited settings [13,15].

Low CD4 cell count and concurrent opportunistic infections were also associated with mortality. This is consistent with evidence linking severe immunosuppression and co-infections to poorer outcomes in cryptococcal meningitis, driven by impaired immune response, higher pathogen burden, and increased risk of complications [15]. In addition, CM-IRIS and anaemia were both associated with increased mortality. The association between CM-IRIS and mortality aligns with evidence from high-burden settings showing increased risk of adverse outcomes following immune reconstitution in patients with cryptococcal infection [14,17]. Anaemia likely reflects advanced disease, comorbidity, and reduced physiological reserve among PLHIV with cryptococcal meningitis [13,18].

These findings suggest that reducing cryptococcal meningitis-related mortality in similar settings will require integrated strategies that prioritise early detection of advanced HIV disease, timely identification and management of intracranial pressure, and strengthened inpatient care for patients with complex comorbidities.

### Study strengths and limitations

This study analysed a large, real-world dataset spanning six years from a national referral hospital in Uganda, providing sufficient temporal coverage and statistical power to estimate inpatient mortality and associated clinical factors among PLHIV with cryptococcal meningitis. The use of routinely collected inpatient data enhances external validity by reflecting actual care practices in a high-burden, resource-limited setting. Inclusion of a broad range of clinical, laboratory, and comorbidity variables enabled a comprehensive assessment of factors associated with mortality, while the high retrieval of medical records and application of multivariable regression strengthened the internal validity of the findings.

However, several limitations should be considered. The retrospective design relied on routine medical records, which limited availability of key management variables, including antifungal treatment regimens, intracranial pressure measurements, and adjunctive therapies; however, standardized data abstraction procedures were applied and analyses were restricted to consistently documented variables. Diagnostic approaches varied between cryptococcal antigen testing and India ink microscopy, which may have introduced misclassification due to differences in sensitivity; analyses were therefore restricted to documented laboratory-confirmed diagnoses. The study was limited to inpatient data, and post-discharge outcomes, including 90-day mortality, could not be assessed; findings were therefore interpreted specifically within the context of in-hospital mortality. The study period overlapped with the COVID-19 pandemic, but available data did not allow assessment of its impact on healthcare access, admission patterns, or inpatient outcomes. Residual confounding may remain due to unmeasured clinical and health-system factors, including variability in treatment practices and timing of care; however, multivariable regression was used to adjust for measured confounders.

## Conclusion

Cryptococcal meningitis accounted for a substantial proportion of HIV-related inpatient admissions and was associated with high in-hospital mortality among adult PLHIV in this setting. Mortality was linked to clinical severity, including neurological manifestations, CM-IRIS, anaemia, low CD4 cell count, and concurrent opportunistic infections. These findings highlight the continued burden of advanced HIV disease and its complications in routine inpatient care. Strengthening early diagnosis, improving clinical monitoring, and optimising inpatient management may contribute to improved outcomes. Further research is needed to evaluate treatment-related and health-system factors influencing survival among PLHIV with cryptococcal meningitis in resource-limited settings.

## Supporting information

S1 File Missingness by variable. (DOCX)

S2 File De-identified dataset analysed for this manuscript. (XLSX)
