# Supplementary material for: Inpatient mortality and associated clinical factors among people living with HIV with cryptococcal meningitis in Uganda: A retrospective cohort study
Source: PLoS One. 2026 Jun 26;21(6):e0340951. doi: 10.1371/journal.pone.0340951 (PMC13308784; doi:10.1371/journal.pone.0340951)
Supplement: S1 File — (DOCX) [file pone.0340951.s001.docx]

**Supplementary material1: Missingness per variable**

| Parameter | Missing (n) | Missing (%) |
| --- | --- | --- |
| **Age (years)** | 0 | 0 |
| **Sex** | 0 | 0 |
| Male | 0 | 0 |
| Female | 0 | 0 |
| **Duration of hospitalization (days)** | 0 | 0 |
| **Duration of symptoms before hospitalization** | 0 | 0 |
| **Duration of ART** | 0 | 0 |
| **Previous history of cryptococcal meningitis** | 0 | 0 |
| **Signs, symptoms and comorbidities present at the time of diagnosis** | 0 | 0 |
| Headache | 0 | 0 |
| Vomiting | 0 | 0 |
| Blurring of vision | 0 | 0 |
| Cryptococcal meningitis IRIS | 0 | 0 |
| Concurrent Opportunistic infections (candidiasis, TB, e.tc) | 0 | 0 |
| Concurrent Diabetes mellitus (DM) | 0 | 0 |
| Concurrent cardiovascular disease (CVDs), hypertension (HTN), etc. | 0 | 0 |
| Concurrent CKD | 0 | 0 |
| Hemoglobin level (g/dl | 0 | 0 |
| CD4 T cell count (cell/uL) | 0 | 0 |
