# Supplementary material for: Inpatient mortality and associated clinical factors among people living with HIV with cryptococcal meningitis in Uganda: A retrospective cohort study
Source: PLoS One. 2026 Jun 26;21(6):e0340951. doi: 10.1371/journal.pone.0340951 (PMC13308784; doi:10.1371/journal.pone.0340951)
Supplement: Attachment — Submitted filename: report_10.64898+2026.01.01.26343321.pdf [file pone.0340951.s003.pdf]

ScreenIT Report

This is an automated report for 10.64898+2026.01.01.26343321.pdf. This report was solicited by the PLOS One editorial team and provided by ScreenIT.

ScreenIT is an independent group of scientists developing automated tools that analyze academic papers. A set of automated tools screened your submitted manuscript and provided the report below. Each tool was created by your academic colleagues with the goal of helping authors.

The tools look for factors that are important for transparency, rigor and reproducibility, and we hope that the report might help you to improve reporting in your manuscript. Within the report you will find links to more information about the items that the tools check. These links include helpful papers, websites, or videos that explain why the item is important.

While our screening tools aim to improve and maintain quality standards they may, on occasion, miss nuances specific to your study type or flag something incorrectly. Each tool has limitations that are described on the ScreenIT website. The tools screen the main file for the paper; they are not able to screen supplements stored in separate files.

Please note that the Academic Editor had access to these comments while making a decision on your manuscript. The Academic Editor may ask that issues flagged in this report be addressed.

If you would like to provide feedback on the ScreenIT tool, please email the team at ScreenIt@bih-charite.de. If you have questions or concerns about the review process, please contact the PLOS One office at plosone@plos.org.

How to read the report:

- no action required

- review for potential issues

- a critical issue was found by a tool

n/a - may not be applicable to your paper

- more information about this item can be obtained using this link

Rigor

✓	Ethics	Field Sample Permit: "Sample size and sampling procedure Sample size estimation followed the formula for unmatched cohort studies described by Kelsey et al. [16], assuming a two-sided significance level of 0.05 and 80% power."
✓	Inclusion and Exclusion Criteria	"Both ARTnaive and ART-experienced patients were eligible, as were referral and non-referral cases."
✓	Flow charts and attrition	Thank you for including a study flow chart and information about attrition and exclusions to help readers evaluate the risk of bias.<br><br>Sentence about attrition: "not required."
✓	Sex as a biological variable	"Males accounted for 52.2% of participants."
✓	Subject Demographics	Age: "Socio-demographic and clinical characteristics The median age of the study population was 36 years (interquartile range [IQR] = 15), with most patients aged 3039 years (38.0%)."
✓	Randomization	"Missing data were assessed for patterns and addressed using multiple imputation by chained equations, under the assumption that data were missing at random."
?	Blinding	Not detected. Please specify whether blinding was used at various phases of the experiment (e.g., blinding of patients, caregivers, outcome assessments, data analysis).
✓	Power Analysis	"Sample size and sampling procedure Sample size estimation followed the formula for unmatched cohort studies described by Kelsey et al. [16], assuming a two-sided significance level of 0.05 and 80% power."
n/a	Replication	not required.

Generated by Barzooka (flow charts), SciScore (everything else).
SciScore only screens the text of the methods section; we apologize if the tool has missed items reported elsewhere in the paper or supplements.

Resources

Persistent unique identifiers for research resources, RRIDs, can be used to track these key resources though the literature. They are requested or required in many journals, but even if your journal does not require them, we strongly encourage the use of these for the readers of papers who wish to easily track down the key materials like antibodies, software tools, or mice.

Software and Algorithms	"Data management and statistical analysis Data were exported from Kobo Collect to Microsoft Excel and analysed using Stata version 15."	Microsoft Excel suggested: Microsoft Excel, RRID:SCR_016137. Please make sure this RRID suggestion is accurate before including in your manuscript.

Generated by SciScore.
SciScore only screens the text of the methods section; we apologize if the tool has missed items reported elsewhere in the paper or supplements.

Transparency

?	Open code	If you wrote code to analyze your data, please consider sharing the code in a public repository. This makes it easier for others to reproduce your analyses, and may also aid others seeking to analyze similar datasets.
?	Open data	If permitted, sharing data on a public repository with a persistent identifier (a DOI or accession number) can improve reproducibility and make it easier for other scientists to expand on your work. Papers with open data are cited more often than papers without open data. Some institutions have an expert who can provide advice on data sharing.
✓	Self-acknowledged limitations	"Strengths and limitations However, several limitations should be considered. The retrospective design relied on routine medical records, which resulted in missing or incomplete documentation for some clinical

		variables, particularly intracranial pressure measurements. Additionally, the single-centre setting may limit generalisability to lower-level facilities, although the hospitals national referral role enhances relevance for similar inpatient contexts. Finally, causal inferences cannot be drawn due to the observational nature of the study."
✓	Conflict of interest statement	ℹ Thank you for stating any conflicts of interest.
✓	Funding statement	ℹ Thank you for reporting your funding source(s), or stating that there was no specific funding for this work.
n/a	Registration statement	ℹ A registered protocol makes the planned research project more transparent and can protect against bias. Please consider registering future confirmatory studies.

Generated by ODDPub, limitation-recognizer, rtransparent, trial-identifier.

Figures

✓	No bar graphs of continuous data	ℹ Thank you for not using bar graphs to present continuous data. Many different datasets and data distributions can lead to the same bar graph, and the actual data may suggest different conclusions from the summary statistics alone.
✓	No rainbow color maps	ℹ Thank you for not using rainbow colormaps. Rainbow color maps distort readers' perception of the data by introducing visual artifacts and are not accessible to colorblind readers.

Generated by JetFighter, Barzooka.
