# Supplementary material for: Inpatient mortality and associated clinical factors among people living with HIV with cryptococcal meningitis in Uganda: A retrospective cohort study
Source: PLoS One. 2026 Jun 26;21(6):e0340951. doi: 10.1371/journal.pone.0340951 (PMC13308784; doi:10.1371/journal.pone.0340951)
Supplement: Attachment — Submitted filename: Response to Reviewers.docx [file pone.0340951.s004.docx]

**Response to Reviewers**

**Reference:** PONE-D-25-68621

**Manuscript title:** Inpatient mortality and associated clinical factors among people living with HIV with cryptococcal meningitis in Uganda: a retrospective cohort study

| **Comment** | **Author Response** | **Location in Revised Manuscript** |
| --- | --- | --- |
| Please ensure that your manuscript meets PLOS ONE's style requirements, including those for file naming. | We carefully reviewed the manuscript against PLOS ONE formatting guidelines. All sections were restructured to align with journal requirements, including standardized headings, figure and table formatting, reference style, and file naming conventions. Minor typographical and formatting inconsistencies were also corrected. | Entire manuscript |
| Clarification is needed regarding methodological details, particularly sampling justification, handling of missing data, and interpretation of associations such as length of hospital stay. | We have expanded the Methods section to clearly describe the total population sampling approach, explaining that all eligible records during the study period were included to maximize statistical power and reduce selection bias. The handling of missing data was revised following verification that no missingness existed; thus, multiple imputation was removed and replaced with complete-case analysis. Additionally, interpretation of the association between length of hospital stay and mortality was refined to avoid causal inference and now emphasizes potential survivorship bias and clinical severity. | Methods (Sampling procedure; Data management; Statistical analysis); Discussion |
| Strengthening the limitations section and refining causal language would significantly improve the manuscript’s clarity and scientific rigor. | The limitations section was substantially expanded to include key methodological constraints, including lack of treatment data, diagnostic heterogeneity, absence of post-discharge outcomes, and potential residual confounding. Throughout the manuscript, causal language was revised and replaced with more appropriate associative wording (e.g., 'associated with' rather than 'led to'), ensuring alignment with observational study design principles. | Discussion; Strengths and Limitations |
| While multiple imputation is mentioned (lines 138-143)... add table showing missingness... | Upon re-evaluation of the dataset, we confirmed that there was no missing data for variables included in the analysis. Therefore, the previously described multiple imputation approach was removed. We explicitly state that complete-case analysis was conducted and provide confirmation in the supplementary dataset. | Methods (Data management); Supplementary file |
| The manuscript provides no information about the actual CM treatment received... | We reviewed patient records for treatment data; however, antifungal regimens were inconsistently documented and could not be reliably analyzed. This has now been explicitly stated in the Methods and Abstract sections. Furthermore, we added this as a key limitation in the Discussion, noting its potential influence on mortality outcomes and interpretation. | Abstract (Methods); Methods; Strengths and Limitations |
| State whether multicollinearity was detected and how it was handled. | We added a detailed description of multicollinearity assessment using variance inflation factors (VIF). Results indicated no multicollinearity concerns (mean VIF = 1.10), and this is now explicitly reported. | Methods (Statistical analysis) |
| CD4 >200 cells/μL is a small reference group... | We revised the CD4 categorization to improve interpretability. The reference category was changed to <50 cells/µL, which is clinically meaningful and more stable. All regression outputs and interpretations were updated accordingly. | Methods; Results; Table 2 |
| Protective effect of 1–2 week symptom duration is inadequately explained. | We refined the interpretation to clarify that this observed association likely reflects survival bias, differences in care-seeking behavior, or disease progression rather than a true protective effect. This explanation has been explicitly added to the Discussion. | Discussion |
| CM-IRIS diagnosis requires definition. | We added a clear operational definition of CM-IRIS in the Methods section, including criteria based on clinical deterioration and timing relative to ART initiation. | Methods |
| What proportion had tuberculosis and other opportunistic infections? | We expanded Table 1 to include a detailed breakdown of opportunistic infections, including tuberculosis and other documented infections, to improve clarity and completeness. | Table 1 |
| In abstract: specify CD4 categories. | The abstract was revised to explicitly reflect CD4 categories (<50 and 50–200 cells/µL), aligning with the main analysis. | Abstract |
| In abstract: condense methods and add treatment findings. | We streamlined the Methods section in the abstract for conciseness and added a statement indicating that treatment data were not consistently available. | Abstract |
| Higher than pooled estimates – provide specific comparison. | We revised this statement to include clearer contextual comparison and appropriate citations, improving interpretability. | Discussion |
| What was the impact of COVID-19? | We added a statement acknowledging that the study period overlapped with the COVID-19 pandemic; however, data limitations prevented formal assessment of its impact. | Strengths and Limitations |
| Expand limitations section. | The limitations section was expanded to include treatment data limitations, diagnostic variability, lack of long-term outcomes, and residual confounding. | Strengths and Limitations |
